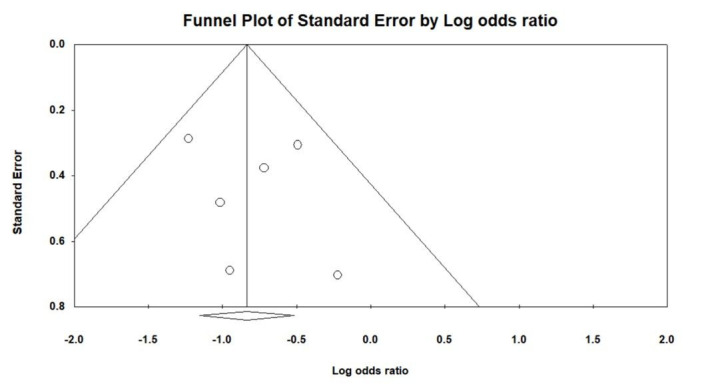# Impact of chronic kidney disease on left atrial appendage occlusion: A meta-analysis of procedural outcomes and complications

**DOI:** 10.1097/MD.0000000000038935

**Published:** 2024-07-19

**Authors:** Wei-Chieh Lee, Wei-Ting Chang, Jhih-Yuan Shih, Po-Jui Wu, Chih-Yuan Fang, Huang-Chung Chen, Yen-Nan Fang, Hsiu-Yu Fang

**Affiliations:** a Division of Cardiology, Department of Internal Medicine, Chi Mei Medical Center, Tainan, Taiwan; b School of Medicine, College of Medicine, National Sun Yat-sen University, Kaohsiung, Taiwan; c Division of Cardiology, Department of Internal Medicine, Kaohsiung Chang Gung Memorial Hospital, Chang Gung University College of Medicine, Kaohsiung, Taiwan.; d Division of Cardiology, Department of Internal Medicine, Jen-Ai Hospital, Taichung, Taiwan

**Keywords:** all-cause mortality, atrial fibrillation, chronic kidney disease, left atrial appendage occlusion, oral anticoagulants

## Abstract

**Background:**

Patients with chronic kidney disease (CKD) experience atrial fibrillation more frequently. The balance of medical management for stroke prevention and bleeding events presents a challenging issue in CKD population. Left atrial appendage occlusion (LAAO) may be an effective solution for stroke prevention in patients who experience frequent bleeding with oral anticoagulants. However, the specific impact of CKD on the procedural success, complications, and outcomes of LAAO implantations remains underexplored.

**Methods:**

We conducted a search of various databases for articles published before October 31, 2023. This search yielded 7 studies, comparing outcomes between CKD and non-CKD cohorts undergoing LAAO implantation. Our analysis focused on CHA2DS2-VASc scores, average eGFR, use of oral anticoagulants, procedural success rates, procedural complications, and associated outcomes.

**Results:**

The meta-analysis included data from 2576 patients, with 1131 identified as having CKD. The CKD group also had higher CHA2DS2-VASc scores (4.7 ± 1.4 vs 4.0 ± 1.5; *P* < .001) and HAS-BLED scores (3.8 ± 1.1 vs 3.1 ± 1.0; *P* < .001) than the non-CKD group. CKD patients showed a nonreduction in procedural success rates and a nonsignificant increase in total complications. The risks of stroke and transient ischemic attack, major bleeding, and cardiovascular mortality were not significantly different between the 2 groups. However, a significantly lower rate of total mortality was observed in the non-CKD group (odds ratio: 0.43; 95% confidence interval, 0.32–0.60).

**Conclusion:**

While CKD is associated with a nonsignificant decrease in procedural success and a nonsignificant increase in complication risks, the outcomes of LAAO implantation are comparably favorable between CKD and non-CKD groups. Despite similar procedural outcomes, the CKD group exhibited a higher rate of all-cause mortality.

## 1. Introduction

Atrial fibrillation (AF), the most common cardiac rhythm disorder, affects 1% to 2% of people and is more prevalent in those with renal insufficiency.^[1–3]^ Chronic kidney disease (CKD), marked by kidney damage and a reduced filtration rate (estimated Glomerular filtration rate below 60 mL/min/1.73 m^2^),^[4]^ heightens the risk of strokes in AF patients, especially as eGFR decreases.^[5,6]^ Stroke prevention in non-dialysis CKD patients mirrors that in the general population. In addition to controlling and detecting carotid atherosclerosis and cerebrovascular disease, the use of direct oral anticoagulant (DOAC) is very important medication for stroke prevention of AF, even though CKD is not included in the CHA2DS2-VASc scoring system.^[7–9]^ DOACs reduce stroke and bleeding risks in AF patients with mild or moderate kidney issues,^[10]^ but their bleeding risk increases in those with CKD due to varying degrees of kidney clearance.^[11–13]^ For AF patients, most clots form in the left atrial appendage (LAA) due to its shape causing blood flow stasis.^[14]^ Therefore, left atrial appendage occlusion (LAAO) has become a key mechanical intervention to prevent AF-related clots, especially for those at high bleeding risk or intolerant to oral anticoagulants (OACs).^[15–17]^ LAAO patients may need less or no OACs, potentially requiring only single antiplatelet therapy. It effectively prevents strokes and minimizes bleeding more than OACs, making it suitable for patients with contraindications to OACs, recurrent bleeding, or previous brain hemorrhages.^[18–21]^ In CKD patients, comorbidities significantly affect the success of coronary interventions and AF ablation,^[22,23]^ but the impact on LAAO implantations remains underexplored.

Recent research has shown that LAAO is equally safe for people with CKD and those without, effectively reducing strokes, transient ischemic attacks (TIA), and bleeding incidents.^[24,25]^ However, some studies have observed a rise in adverse events among CKD patients.^[26]^ This increase is linked to higher mortality rates, primarily due to the elevated cardiovascular (CV) risk in CKD individuals.^[27–29]^ Notably, one study pointed out a greater likelihood of thromboembolic and bleeding issues in those with CKD.^[30]^ Considering the inconsistencies in previous studies, our meta-analysis aims to rigorously evaluate the effectiveness and safety of LAAO in both CKD and non-CKD populations.

## 2. Methods

### 2.1. Search strategies, trial selection, quality assessment, and data extraction

Two cardiologists, Wei-Chieh Lee and Hsiu-Yu Fang, conducted a comprehensive literature search in various databases, including PubMed, Embase, ProQuest, ScienceDirect, Cochrane Library, ClinicalKey, Web of Science, and ClinicalTrials.gov. They searched for articles published up to October 31, 2023, using key terms such as “atrial fibrillation or AF,” “left atrial appendage occlusion or left atrial appendage occlude or LAAO,” “chronic kidney disease or CKD,” and “oral anticoagulants.” The search was conducted without language restrictions to broaden the scope of relevant studies. In instances of disagreement, a third reviewer, Po-Jui Wu, was consulted for resolution. This analysis included randomized controlled studies and observational studies that compared the efficacy and safety outcomes of LAAO in groups with and without CKD. The inclusion criteria specified human studies with a parallel design, focusing on the comparison of efficacy and safety of LAAO between CKD and non-CKD groups. Exclusion criteria encompassed case reports or series, animal studies, review articles, conference abstracts, and unpublished data. No language limitations were set, aiming to increase the pool of eligible articles. Supplemental Figure 1, Supplemental Digital Content, http://links.lww.com/MD/N203, a PRISMA flow chart, depicts the literature search and screening protocol.

### 2.2. Outcomes

The outcomes of interest in this study included both short-term and long-term results. Short-term outcomes encompassed procedural success, periprocedural stroke, cardiac tamponade, and overall complications. Long-term outcomes focused on stroke/TIA major bleeding events, CV mortality, and all-cause mortality.

### 2.3. Statistical analysis

We extracted the frequency of each assessed outcome from the included studies and presented the data as cumulative rates. A random effects model was utilized to aggregate individual odds ratios (ORs), with all analyses conducted using the Comprehensive Meta-Analysis software version 3 (Biostat, Englewood, NJ). To evaluate heterogeneity among the trials, the chi-square test was applied, considering *P* ≤ .10 as indicative of significant heterogeneity. We also used the *I*^2^ statistics, categorizing heterogeneity as low to high ranging from 25% to 75%. To determine potential publication bias, we employed Egger test, visualizing the results through funnel plots, with statistical significance defined at *P* ≤ .10. For comparisons between CKD and non-CKD groups, statistical significance was established at *P* < .05.

## 3. Results

### 3.1. Characteristics of included studies

The process of selecting studies for inclusion is illustrated in Supplemental Figure 1, Supplemental Digital Content, http://links.lww.com/MD/N203. A total of 7 studies^[24–30]^ met the inclusion criteria, encompassing 2576 participants. Details regarding the study period, participant characteristics, criteria for CKD, and the follow-up period are outlined in Table 1.

**Table 1:** Characteristics of the 7 included studies.

First author (yr)	Patients number (male %)	Age (yr)	Study period	Comparison of groups	The criteria of CKD	Follow-up	Reference number
Kefer et al (2016)	630 (62)	75 ± 8	December 2008 to November 2013	CKD (375) vs non-CKD (639)	eGFR < 60 mL/min/1.73 m^2^	498 d (186–753)	^[24]^
Xue et al (2018)	203 (68)	75 ± 8	February 2012 to January 2017	CKD (151) vs non-CKD (149)	eGFR < 60 mL/min/1.73 m^2^	637 ± 398 d	^[25]^
Brockmeyer et al (2019)	146 (58)	77 ± 7	March 2012 to March 2016	CKD (81) vs non-CKD (65)	eGFR < 60 mL/min/1.73 m^2^	391 ± 309 d	^[26]^
Fastner et al (2021)	623 (61)	77 ± 3	July 2014 to January 2016	CKD (299) vs non-CKD (324)	eGFR < 60 mL/min/1.73 m^2^	365 d	^[27]^
Michlicka-Kłyś et al (2022)	272 (56)	73 ± 9	January 2009 to December 2019	CKD (105) vs non-CKD (167)	eGFR < 60 mL/min/1.73 m^2^	25.56 ± 17.9 mo	^[28]^
Benini Tapias et al (2022)	124 (62)	75 ± 4	January 2011 to July 2019	CKD (71) vs non-CKD (53)	eGFR < 60 mL/min/1.73 m^2^	567 d (207–944)	^[29]^
Lind et al (2023)	58 (60)	74 ± 8	January 2011 to January 2017	CKD (49) vs non-CKD (48)	eGFR < 60 mL/min/1.73 m^2^	36 mo	^[30]^

CKD = chronic kidney disease, eGFR = estimated Glomerular filtration rate.

### 3.2. Patient demographics

Table 2 describes a comprehensive comparison of patient demographics between the non-CKD and CKD groups, encompassing 2576 participants divided into 1445 non-CKD and 1131 CKD patients. The data highlight key demographic differences, with the average age significantly higher in the CKD group (78 ± 6.3 years) compared to the non-CKD group (74 ± 7.4 years, *P* < .001). The percentage of males was also lower in the CKD group (55.1%) compared to the non-CKD group (66.4%, *P* < .001). Notable differences were observed in the prevalence of diabetes mellitus (39.6% in CKD vs 24.5% in non-CKD, *P* < .001), hypertension (89.3% in CKD vs 86.2% in non-CKD, *P* = .025), and heart failure (31.0% in CKD vs 18.5% in non-CKD, *P* < .001). Coronary artery disease was also more prevalent in the CKD group (51.6% vs 33.3% in non-CKD, *P* < .001), while the incidence of prior stroke was lower (26.0% in CKD vs 33.4% in non-CKD, *P* < .001). The CHA2DS2-VASc score was higher in the CKD group (4.7 ± 1.4) compared to the non-CKD group (4.0 ± 1.5, *P* < .001), as was the HAS-BLED score (3.8 ± 1.1 in CKD vs 3.1 ± 1.0 in non-CKD, *P* < .001). The eGFR was significantly lower in the CKD group (42.2 ± 13.6 mL/min/1.73 m^2^) compared to the non-CKD group (83.9 ± 18.9 mL/min/1.73 m^2^, *P* < .001). These demographic differences are crucial for understanding the diverse impacts of LAAO in patients with and without CKD.

**Table 2:** Patients’ demographics between non-CKD and CKD populations.

	Non-CKD	CKD	*P* value
Number	1445	1131	
Age (yr)	74 ± 7.4 (1445)	78 ± 6.3 (1131)	<.001
Male sex (%)	66.4 (960/1445)	55.1 (623/1131)	<.001
Diabetes mellitus (%)	24.5 (342/1397)	39.6 (429/1082)	<.001
Hypertension (%)	86.2 (1102/1278)	89.3 (916/1026)	.025
Permanent AF (%)	54.2 (657/1213)	53.8 (508/945)	.853
Heart failure (%)	18.5 (258/1397)	31.0 (335/1082)	<.001
Coronary artery disease (%)	33.3 (432/1296)	51.6 (506/980)	<.001
Prior stroke (%)	33.4 (467/1397)	26.0 (281/1082)	<.001
Prior major bleeding (%)	42.4 (521/1230)	43.3 (423/977)	.671
CHA2DS2-VASc score	4.0 ± 1.5 (1445)	4.7 ± 1.4 (1131)	<.001
HAS-BLED score	3.1 ± 1.0 (1445)	3.8 ± 1.1 (1131)	<.001
eGFR (mL/min/1.73^2^)	83.9 ± 18.9 (1121)	42.2 ± 13.6 (832)	<.001
On anticoagulant (%)	32.5 (351/1081)	38.3 (316/826)	.009

Data are expressed as mean ± standard deviation or as number (percentage).

AF = atrial fibrillation, CKD = chronic kidney disease, eGFR = estimated Glomerular filtration rate.

### 3.3. Pooled OR of short-term outcomes

**Figure 1.**
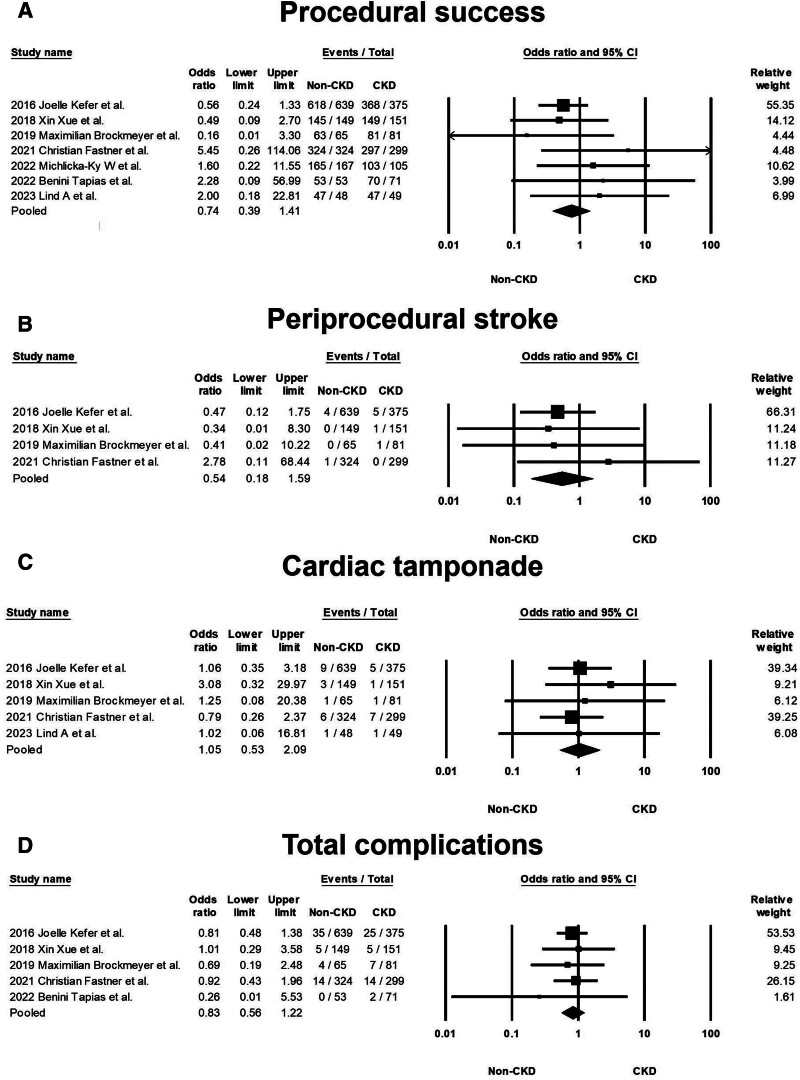
(A) Forest plots demonstrating the rate of procedural success between the non-CKD and CKD groups in 7 studies. (B) Forest plots demonstrating the risk of periprocedural stroke between the non-CKD and CKD groups in 4 studies. (C) Forest plots demonstrating the risk of cardiac tamponade between the non-CKD and CKD groups in 5 studies. (D) Forest plots demonstrating the risk of complications between the non-CKD and CKD groups in 5 studies. Abbreviation: CKD: chronic kidney disease. CKD = chronic kidney disease.

Figure 1 illustrates the procedural outcomes for patients with and without CKD. The analysis reveals a nonsignificant trend toward reduced procedural success in the CKD group (OR 0.74, 95% CI 0.39–1.41), exhibiting low heterogeneity (Cochran *Q* = 4.983; df = 6; *I*^2^ = 0%; *P* = .546). Egger test indicates no significant publication bias for the overall OR of procedural success (*t* = 1.305; df = 5; *P* = .249), as shown in Supplemental Figure 2, Supplemental Digital Content, http://links.lww.com/MD/N202. There was no significant difference in the risk of periprocedural stroke between the groups (OR 0.54, 95% CI 0.18–1.59), with similarly low heterogeneity (Cochran *Q* = 1.164; df = 3; *I*^2^ = 0%; *P* = .762), and a nonsignificant publication bias (*t* = 0.533; df = 2; *P* = .647) as per Supplemental Figure 3, Supplemental Digital Content, http://links.lww.com/MD/N204. The likelihood of cardiac tamponade was consistent between the groups, with a pooled OR of 1.05 (95% CI 0.53–2.09) and low heterogeneity (Cochran *Q* = 1.139; df = 4; *I*^2^ = 0%; *P* = .888). The associated publication bias was nonsignificant (*t* = 1.208; df = 3; *P* = .314), detailed in Supplemental Figure 4, Supplemental Digital Content, http://links.lww.com/MD/N205. The overall complication rates showed no significant difference (OR 0.83, 95% CI 0.56–1.22), with low heterogeneity (Cochran *Q* = 0.805; df = 4; *I*^2^ = 0%; *P* = .938) and a nonsignificant publication bias (*t* = 1.141; df = 3; *P* = .337) as depicted in Supplemental Figure 5, Supplemental Digital Content, http://links.lww.com/MD/N206. These findings indicate no statistically significant disparities in procedural success or complication rates between patients with and without CKD, as evidenced by the confidence intervals of all pooled ORs crossing the line of no effect (OR = 1).

### 3.4. Pooled OR of long-term outcomes

**Figure 2.**
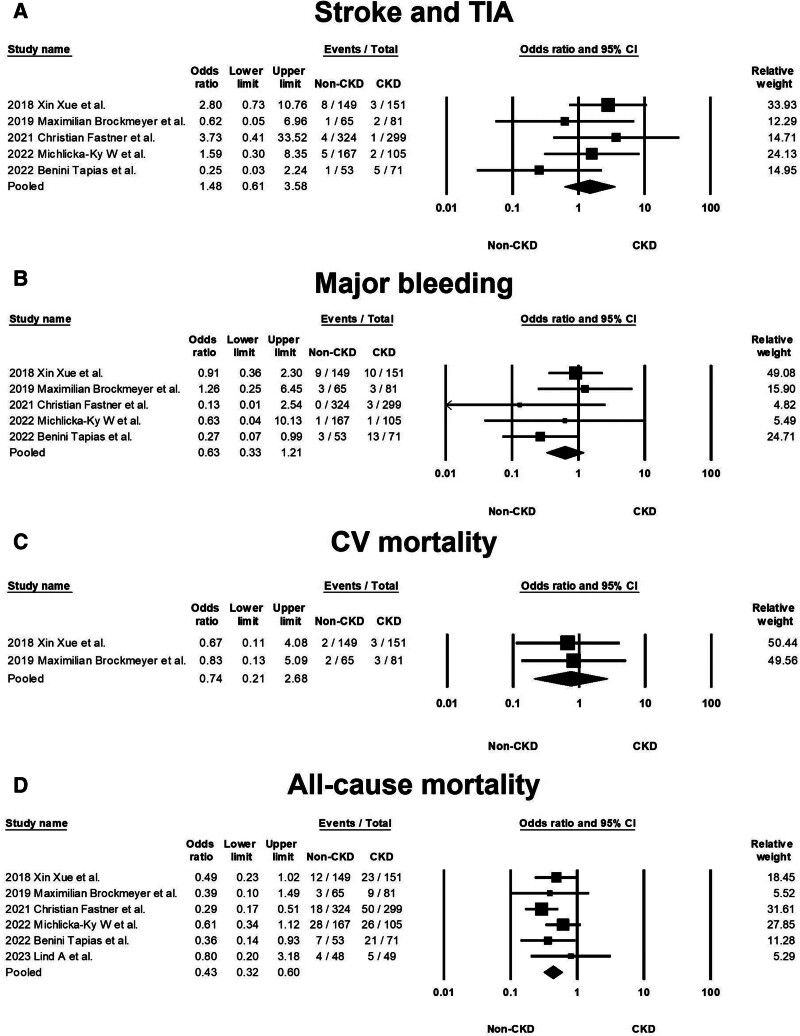
(A) Forest plots demonstrating the risk of stroke and TIA between the non-CKD and CKD groups in 5 studies. (B) Forest plots demonstrating the risk of major bleeding events between the non-CKD and CKD groups in 5 studies. (C) Forest plots demonstrating the risk of cardiovascular mortality between the non-CKD and CKD groups in 2 studies. (D) Forest plots demonstrating the risk of all-cause mortality between the non-CKD and CKD groups in 6 studies. CKD = chronic kidney disease, TIA = transient ischemic attack, CV = cardiovascular.

Figure 2 evaluates the long-term outcomes between non-CKD and CKD groups. The pooled OR for stroke and TIA is 1.48, with a 95% CI of 0.61 to 3.58, indicating no statistically significant difference between the groups, and demonstrating low heterogeneity (Cochran *Q* = 4.558; df = 4; *I*^2^ = 12.24%; *P* = .336). No significant publication bias was detected regarding stroke and TIA (*t* = 1.258; df = 3; *P* = .298), as shown in Supplemental Figure 6, Supplemental Digital Content, http://links.lww.com/MD/N207. The pooled OR for major bleeding is 0.63 (95% CI 0.33–1.21), showing no significant difference between non-CKD and CKD patients, with negligible heterogeneity (Cochran *Q* = 3.993; df = 4; *I*^2^ = 0%; *P* = .407) and no significant publication bias (*t* = 0.780; df = 3; *P* = .492), detailed in Supplemental Figure 7, Supplemental Digital Content, http://links.lww.com/MD/N208. For CV mortality, the pooled OR is 0.74 (95% CI 0.21–2.68), with low heterogeneity (Cochran *Q* = 0.025; df = 1; *I*^2^ = 0%; *P* = .874). The pooled OR for all-cause mortality stands at 0.43 (95% CI 0.32–0.60), suggesting a trend toward lower risk in the non-CKD group, accompanied by low heterogeneity (Cochran *Q* = 4.140; df = 5; *I*^2^ = 0%; *P* = .529) and no significant publication bias (*t* = 0.534; df = 4; *P* = .621), as per Supplemental Figure 8, Supplemental Digital Content, http://links.lww.com/MD/N209.

These findings collectively indicate that apart from a trend indicating lower all-cause mortality in non-CKD patients, there were no significant disparities in the long-term outcomes of stroke/TIA, major bleeding, and CV mortality between the 2 groups. The confidence intervals for these outcomes include the line of no effect (OR = 1), reinforcing the lack of significant differences.

## 4. Discussion

This study compared outcomes between non-CKD and CKD populations in AF patients undergoing LAAO, aiming to explore the impact of CKD on the efficacy and safety of LAAO in these groups. The CKD population exhibited a higher prevalence of comorbidities, as well as increased CHA2DS2-VASc and HAS-BLED scores. The average age in the study population was 76 ± 7.2 years, with 30.2% having a history of stroke, 42.8% experiencing major bleeding previously, but only 35.0% of the patients had been on OACs. Between the non-CKD and CKD groups, CKD was associated with a nonsignificant reduction in procedural success and a nonsignificant increase in complication risks. Post-LAAO implantation, the incidence of stroke/TIA and major bleeding was similar between the 2 groups, despite a higher potential for stroke in the CKD group according to significantly higher CHA2DS2-VASc scores (non-CKD vs CKD; 4.0 ± 1.5 vs 4.7 ± 1.4; *P* < .001). However, a higher incidence of all-cause mortality was observed in the CKD population, which may be attributed to their complex baseline characteristics.

Based on 7 studies^[24–30]^ that we reviewed, there were no significant differences in procedural success and complications between the non-CKD and CKD groups. Previous research^[24–26,28]^ indicates that LAAO significantly reduces the rates of stroke/TIA and major bleeding across all stages of CKD, compared to the expected annual risk. Notably, the cessation of anticoagulant therapy following LAAO was particularly beneficial for patients at a very high risk of bleeding, where a significant reduction in major bleeding events was observed. In our study, the CKD population exhibited a higher predicted bleeding risk according to the HAS-BLED score (non-CKD vs CKD; 3.1 ± 1.0 vs 3.8 ± 1.1; *P* < .001), yet they showed similar rates of long-term major bleeding. Consequently, LAAO plays a crucial role in significantly reducing bleeding events in the CKD population, especially for those with a higher bleeding risk and who are intolerant to OACs.

Previous meta-analyses have not standardized the criteria and definition of CKD, often including patients with end-stage renal disease (ESRD).^[31]^ In contrast, our study defined CKD as an eGFR of <60 mL/min/1.73 m² and excluded patients with ESRD. Patients undergoing dialysis face significant constraints regarding OAC use. Currently, there is no uniform guideline for anticoagulation following LAAO, and the practice of using post-procedural dual antiplatelet agents (excluding OACs) after LAAO is common.^[32]^ Three of the studies we included provided detailed regimens of post-procedural OAC or antiplatelet agents.^[24,27,28]^ Other important issue is device-related thrombosis after LAAO. Device-related thrombosis following LAAO is linked to ischemic events, and renal insufficiency increases the risk of such thrombosis by approximately fourfold.^[33, 34]^ Therefore, there is a significant need for larger studies to assess the long-term risk of ischemic events following LAAO in CKD patients, particularly those not protected by OACs or on low-dose OACs.

## 5. Limitations

This study is subject to several limitations. First, most of the included studies are observational cohort studies, which introduces a potential for selection bias. However, these studies collectively encompass 2576 participants, including the most recent research. Second, we were unable to access complete baseline characteristics for all participants in the studies we reviewed. Third, this meta-analysis did not analyze the post-procedural regimen of OAC or antiplatelet therapy, nor the incidence of device-related thrombosis after LAAO. Fourth, the studies included in our review had varying follow-up periods. Despite these limitations, this study provides valuable insights into the efficacy and safety of LAAO in the CKD population.

## 6. Conclusion

While CKD is associated with a slight, nonsignificant increase in complication risks and a marginal decrease in procedural success, the outcomes of LAAO are similarly favorable in both CKD and non-CKD groups. Despite higher rates of all-cause mortality and more comorbidities in the CKD group, LAAO shows comparable effectiveness in reducing stroke/TIA and major bleeding incidents when compared to the non-CKD population.

## Author contributions

**Conceptualization:** Wei-Chieh Lee.

**Data curation:** Wei-Chieh Lee, Po-Jui Wu.

**Formal analysis:** Wei-Chieh Lee, Huang-Chung Chen.

**Investigation:** Wei-Chieh Lee, Po-Jui Wu.

**Writing – original draft:** Wei-Chieh Lee.

**Validation:** Wei-Ting Chang, Jhih-Yuan Shih.

**Visualization:** Wei-Ting Chang, Jhih-Yuan Shih, Chih-Yuan Fang, Huang-Chung Chen, Yen-Nan Fang.

**Software:** Po-Jui Wu.

**Writing – review & editing:** Hsiu-Yu Fang.

## Supplementary Material

**Figure:**
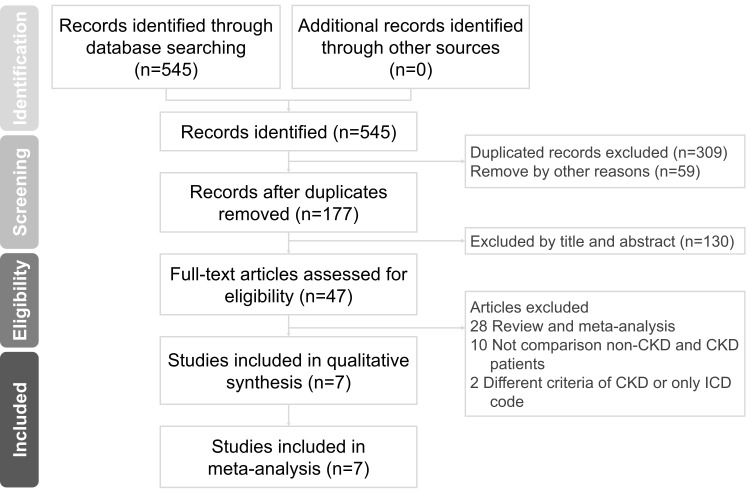


**Figure:**
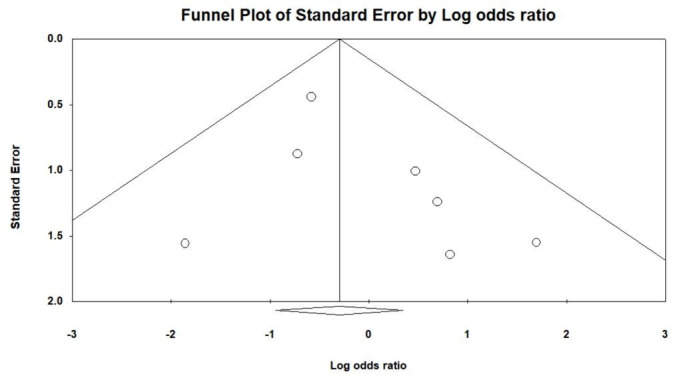


**Figure:**
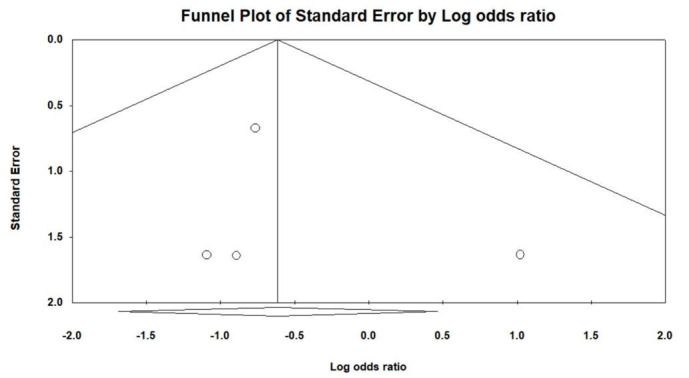


**Figure:**
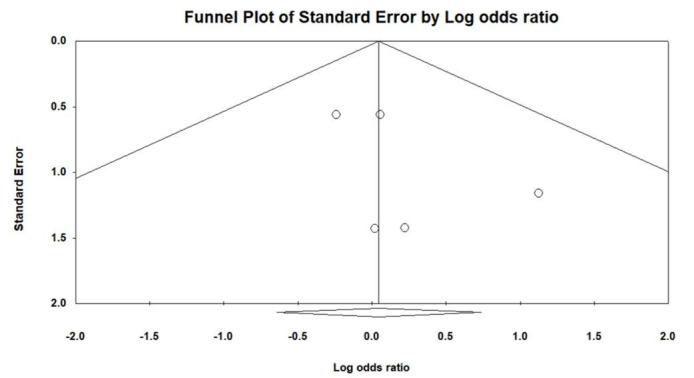


**Figure:**
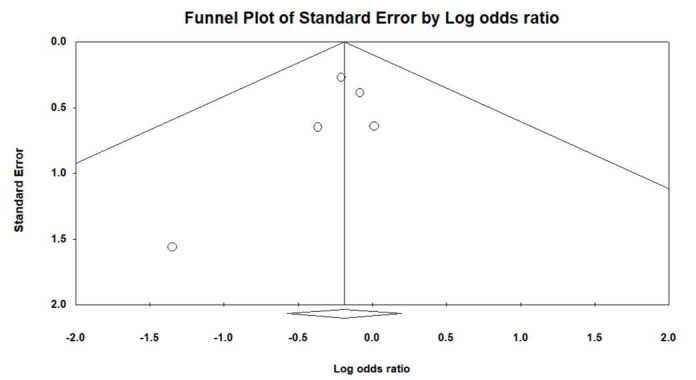


**Figure:**
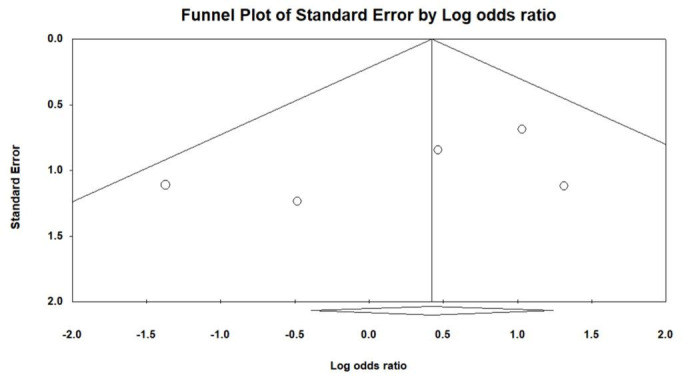


**Figure:**
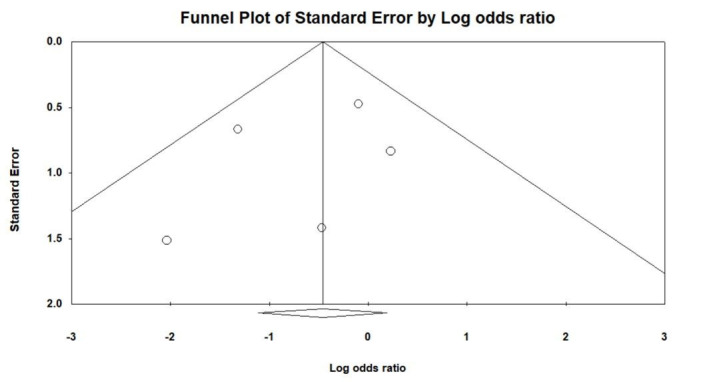


**Figure:**